# Application of Heparin Affinity Chromatography to Produce a Differential Vaccine without Eliciting Antibodies against the Nonstructural Proteins of the Serotype O Foot-and-Mouth Disease Viruses

**DOI:** 10.3390/v12121405

**Published:** 2020-12-07

**Authors:** Sun Young Park, Jung-Min Lee, Ah-Young Kim, Sang Hyun Park, Jae-Seok Kim, Hyejin Kim, Jung-Won Park, Jong-Hyeon Park, Young-Joon Ko, Choi-Kyu Park

**Affiliations:** 1 Animal and Plant Quarantine Agency, 177 Hyeoksin-8-ro, Gimcheon-si 39660, Gyeongsangbuk-do, Korea; sun3730@korea.kr (S.Y.P.); wjdalslee@korea.kr (J.-M.L.); mochsha@korea.kr (A.-Y.K.); shpark0205@korea.kr (S.H.P.); kimjs0728@korea.kr (J.-S.K.); hyejin86@korea.kr (H.K.); parkjw6254@korea.kr (J.-W.P.); parkjhvet@korea.kr (J.-H.P.); 2 Animal Disease Intervention Center, College of Veterinary Medicine, Kyungpook National University, Daegu 41566, Gyeongsangbuk-do, Korea

**Keywords:** foot-and-mouth disease virus, vaccine, nonstructural protein, purification, chromatography

## Abstract

Although polyethylene glycol (PEG) application is the most widely used method in removing nonstructural proteins (NSPs) for foot-and-mouth disease (FMD) vaccine production, some NSPs remaining in the antigen could elicit antibodies against these proteins after repeated vaccinations in livestock. Therefore, the purpose of this study was to purify the FMD virus (FMDV) via affinity chromatography using a heparin ligand to remove most proteins, including NSPs. Chromatography showed an intact virus (146S) particle recovery of 70% or more for three different strains of serotype O FMDV (two locally isolated strains and one genetically modified strain). The experimental vaccine made with antigens eluted via heparin affinity chromatography elicited virus-neutralizing antibodies against homologous viruses but did not induce antibodies against NSPs even after five immunizations in goats; this indicated that the NSPs were effectively removed from the vaccine antigen. This method can then be used to produce a higher-quality vaccine compared with PEG application in terms of the purity of the FMD vaccine. Therefore, this result would be an important groundwork for advanced FMD vaccine manufacturing in the near future.

## 1. Introduction

Foot-and-mouth disease (FMD) is a highly contagious vesicular disease affecting cloven-hoofed animals and often causes enormous economic loss in the livestock industry [1].

The FMD virus (FMDV), the causative agent of FMD, belongs to the *Aphthovirus* genus of the *Picornaviridae* family [2]. It has a positive-sense, single-stranded RNA genome that is translated into a polyprotein, which is further cleaved into structural proteins (SPs) and nonstructural proteins (NSPs) [3,4,5].

The Republic of Korea has experienced 11 FMD outbreaks since 2000 [6]. To prevent annual outbreaks, the Korean government implemented a nationwide vaccination policy after an extensive FMD outbreak during 2010–2011. It has imported FMD vaccines annually from Argentina, the UK, and Russia and has therefore set out to develop a domestic FMD vaccine using local virus isolates [7].

Unlike other animal vaccines, a virus purification step should be included in FMD vaccine production to remove NSPs. Since NSP antibodies are only detected in animals infected with FMDV, it is considered an indicator to differentiate between FMDV naturally infected and vaccinated animals. Therefore, only high-purity FMD vaccines without NSP could allow differential diagnosis of FMD to monitor virus circulation. Although polyethylene glycol (PEG) application is the most widely used method in removing NSPs for FMD vaccine production, some NSPs left in the antigen, made using this method, could elicit NSP antibodies after repeated vaccination in animals [8,9]. In addition, we reported that NSPs could be efficiently removed by chloroform [10]. However, this method does not purify the FMDV, and many proteins derived from cells are left in the samples. Unwanted cellular protein contaminants can cause an allergic reaction in animals after multiple vaccinations [11].

In this regard, we attempted to purify the FMDV via affinity chromatography with high purity using a heparin ligand. The FMDV has been reported to be recognized by two classes of receptors, i.e., integrin and heparan sulfate. Integrin is mainly employed when FMDV infect the animals and interact with the Arg-Gly-Asp (RGD) sequence in the GH loop of the FMDV VP1 region [12,13]. However, under the conditions of adapted cell culture, FMDV obtains the ability to utilize heparan sulfate as its receptor [12]. Heparan sulfates are negatively charged disaccharide repeating polymers of iduronic acid and glucosamine [14]. They are found as the carbohydrate component of many proteins called heparan sulfate proteoglycans. The heparan sulfate receptor binding site is located 15 Å away from the RGD motif in the reduced conformation of the FMDV [14]. In the production of FMD vaccine antigens, FMDV is cultured in baby hamster kidney 21 (BHK-21) cells, thus has an affinity for heparin that is structurally similar to heparan sulfate receptor.

Therefore, the purpose of this study was to purify FMDV by heparin affinity chromatography without nonessential proteins, including NSPs, and the purity of the FMD vaccine prepared with purified FMDV was verified by evaluating NSP antibodies after repeated immunization in goats.

## 2. Materials and Methods

### 2.1. Ethic Statement

The animal experiments in this study were approved by the Institutional Animal Care and Use Committee (IACUC) and conducted in appliance with the guidelines of laboratory animal welfare and ethic of the animals in the Animal and Plant Quarantine Agency in Republic of Korea (IACUC Approval No. 2020-542, Project identification No. 2020-428).

### 2.2. Cells and Viruses

BHK-21 suspension cells were developed from the original adherent cell line, BHK-21 (C-13) (ATCC, Manassas, VA, USA) by the Animal and Plant Quarantine Agency and the Korea Research Institute of Bioscience & Biotechnology for use in suspension culture with serum-free media [15]. ProVero™ 1 cell culture medium (Lonza, Basel, Switzerland), a serum-free cell culture medium, was used to culture the BHK-21 suspension cells by incubation at 110 rpm in a shaking incubator at 37 °C with 5% CO_2_. In this study, three different strains of serotype O FMDV were used. Two strains were domestic isolates called FMDV O/Boeun/SKR/2017 and FMDV O Jincheon/SKR/2014. The other strain was a genetically engineered virus called the FMDV Om-O-PanAsia2 recombinant strain that was made from a recombination of the backbone region of the O1 Manisa/Turkey/69 strain with the P1 region only being replaced by the O PAK/44/2008 strain [16].

### 2.3. Virus Propagation

The BHK-21 suspension cells started to grow at a seeding density of 3 × 10^5^ cells/mL and reached at least 1.5 × 10^6^ cells/mL within 72 h. The serotype O FMDV strains were inoculated into the BHK-21 suspension cells (3 × 10^6^ cells/mL) at a multiplicity of infection of 0.001 on a shaking platform in an incubator at 37 °C with 5% CO_2_. Viruses were harvested at 16 h post-infection and clarified by centrifugation at 3000× *g* for 20 min at 4 °C to remove cell debris. Binary ethylenimine (BEI; Sigma-Aldrich, St. Louis, MO, USA) was added at 3 mM to the virus culture supernatant to inactivate the FMDV, and the mixture was incubated at 100 rpm for 28 h at 26 °C. Subsequently, the BEI was neutralized by adding 1 M sodium thiosulfate (Daejung Chemicals & Metals, Siheung, Korea) at a final concentration of 2% (*v*/*v*).

### 2.4. Heparin Affinity Chromatography

The AKTA Pure 150 fast protein liquid chromatography system (GE Healthcare, Uppsala, Sweden) connected to a fraction collector F9-R (GE Healthcare) was used for the liquid chromatography experiments. The chromatography system was controlled using AKTA Explorer via the Unicon version 6.4 program (GE Healthcare). Prior to column purification, the inactivated FMDV supernatant was concentrated 10-fold and exchanged with a column binding buffer (20 mM Tris-HCl, 300 mM KCl, pH 7.6) for desalting using a 10-kDa cutoff Centricon Plus-70 centrifugal unit (Merck Millipore, Billerica, MA, USA). The three strains of serotype O FMDV culture supernatant was loaded onto a HiTrap HP Heparin column (7 mm × 25 mm, column volume [CV] = 1.0 mL, GE Healthcare) at a flow rate of 1.0 mL/min. Before sample loading, the column was equilibrated with binding buffer. After loading the 2 CV of the sample, 10 CV of binding buffer was flowed through the column to wash out the remaining unbound proteins. The flow-through and washing fractions containing proteins unbound to the heparin ligand were collected to determine the recovery rates of the FMDV and total protein contents. The FMDV adsorbed on the heparin ligand was gradient-eluted with a binding buffer containing 0.3–1 M KCl. The samples were traced by UV monitoring at 280 nm and collected from the peak fraction during the elution step were pooled and stored at −70 °C until use.

### 2.5. Total Protein Assay

The BCA protein assay kit (Thermo Fisher Scientific, Waltham, MA, USA) was used to measure the total proteins in each sample. The total protein content was measured in accordance with the manufacturer’s instructions with bovine serum albumin as a standard, and the absorbance was read at 562 nm.

### 2.6. Quantification of FMDV Particles (146S)

The samples (virus supernatant and flow-through/elution fraction from chromatography) were treated with chloroform (Merck KGaA, Darmstadt, Germany) and benzonase nuclease (Merck KGaA) to reduce background signals induced by protein/nucleic acid-derived impurities. First, 50% (*v*/*v*) chloroform was added to the samples and mixed by inverting for 5 min, and the aqueous layer was then harvested by centrifugation at 3000× *g* for 15 min. Thereafter, these samples were treated with benzonase nuclease at a concentration of 25 units/mL and mixed at 37 °C for 1 h. After centrifugation at 10,000× *g* for 30 min, the supernatant was harvested. The pretreated samples were then layered onto 15–45% sucrose density gradients (SDG) and ultra-centrifuged again at 100,000× *g* for 4 h at 4 °C using an SW41Ti rotor. The ultra-centrifuged SDG was fractionated using a continuous density gradient fractionator (Teledyne ISCO, Lincoln, NE, USA) [17], and the absorbance of each fraction at 254 nm was consecutively recorded by the spectrophotometer component of the instrument. The area under the peak for specific fractions was measured to calculate the quantity of 146S (µg/mL) according to the formula presented in a previous study [17].

### 2.7. SDS-PAGE, Western Blot Analysis

The protein samples of each fraction obtained in the purification process were subjected to sodium dodecyl sulfate polyacrylamide gel electrophoresis (SDS-PAGE). The protein samples were mixed with 4× non-reducing lithium dodecyl sulfate sample buffer (Invitrogen, Carlsbad, CA, USA) and 10× reducing agent (1M dithiothreitol; Merck KGaA). The pretreated samples were heated at 70 °C for 10 min and run on 4–12% Bis-Tris gradient gels (Invitrogen). The gel was stained by Coomassie Brilliant Blue R-250 staining solution (Biorad, Hercules, CA, USA) for 1 h with gentle agitation. The stained gel was then treated with the destaining solution (Biosesang, Sungnam, Korea) and agitated until the protein bands were visible. For Western blot analysis, the gel was transferred to a nitrocellulose membrane using the iBlot 2 gel-transfer device (Invitrogen). The membranes were blocked with 5% skimmed milk in phosphate-buffered saline with Tween-20 (PBST; 10 mM sodium phosphate, 132 mM NaCl, 2.7 mM KCl, and 0.05% Tween-20, pH 7.4). The membranes were washed and incubated with anti-FMDV 3B monoclonal antibody (4G24, supplied by the Median Diagnostics Inc. (Chuncheon, Republic of Korea) and diluted 1/2000 in PBST). After washing the membranes, they were incubated with goat anti-mouse horseradish peroxidase-conjugated secondary antibodies (Invitrogen) diluted 1/4000 in PBST. The proteins were visualized with Pierce ECL Substrate (Invitrogen) using the Azure C600 imaging system and the cSeries Capture Software (Azure Biosystem, Dublin, CA, USA).

### 2.8. Transmission Electron Microscopy (TEM)

The fractions eluted with a high concentration of salt from the heparin ligand were collected and concentrated to the same volume that was initially loaded onto a HiTrap HP Heparin column using a 10-kDa cutoff Centricon Plus-70 centrifugal unit (Merck Millipore). It was layered on top of sucrose density gradients and then centrifuged at 100,000× *g* for 4 h. The band between the 30% and 35% sucrose layers was collected and centrifuged at 100,000× *g* for 4 h. The resulting pellet was resuspended and dialyzed with Tris-KCl buffer (20 mM Tris-HCl, 300 mM KCl, pH 7.6) at 4 °C to eliminate residual sucrose. One drop of the purified FMDV suspension was placed on formvar-coated grids and negatively stained with 1% uranyl acetate. The morphology of purified FMDV particles were examined via TEM (Hitachi 7100; Hitachi, Tokyo, Japan).

### 2.9. Preparation of Vaccines

The experimental vaccines were prepared with the FMDV O/Boeun/SKR/2017 strain via: (i) PEG-precipitation; and (ii) heparin affinity chromatography. For the first group, the FMDV culture supernatant was treated with 7.5% (*w/v*) PEG 6000 (Sigma-Aldrich), stirred overnight at 4 °C, and centrifuged at 10,000× *g* for 30 min. After the supernatant was removed, the pellet was resuspended in Tris-KCl buffer (20 mM Tris-HCl, 300 mM KCl, pH 7.6) and adjusted to a concentration of 15 μg/mL. For the second group, the heparin column eluted antigens were concentrated to 15 μg/mL using a 10-kDa cutoff Centricon Plus-70 centrifugal unit (Merck Millipore). Saponin (Sigma-Aldrich) and aluminum hydroxide gel (General Chemical, Parsippany, NJ, USA) were added to these two kinds of antigens, and the ISA 206 VG adjuvant (Seppic, Paris, France) pre-warmed at 30 °C was then added in a ratio of 1:1. After the mixtures were incubated at 20 °C for 1 h in a water bath with blocked light, they were stored at 4 °C until use.

### 2.10. Animal Experiments

Korean black goats at the age of 8 months, which were not previously FMD-vaccinated, were tested via enzyme-linked immunosorbent assay (ELISA) to confirm negativity for SP and NSP antibodies. Twenty goats were injected intramuscularly with vaccines containing PEG-treated antigen (*n* = 10) and heparin affinity column purified antigen (*n* = 10). All goats were vaccinated five times every 4 weeks at a dose of 15 μg/2 mL and bled at an interval of 4 weeks after vaccination to obtain serum samples. 

### 2.11. ELISA

PrioCHECK FMDV NSP and serotype O SP antibody detection kits (Prionics, Lelystad, The Netherlands) were used to measure the antibody levels elicited by the vaccines. The competition ELISAs were performed in accordance with the manufacturer’s instructions. Test sera were added to antigen-coated plates and incubated for 1 h at 25 °C. After the plates were washed, horseradish peroxidase-conjugated monoclonal antibodies against the immobilized FMDV antigen (3ABC (NSP) and inactivated serotype O FMDV (SP)) were added to the plates, followed by incubation for 1 h. Finally, the plates were washed and 3,3,5,5-tetramethylbenzidine substrate solution was added. The colorimetric reaction was stopped with sulfuric acid solution after 15 min, and the optical density was measured at 450 nm. The ELISA results were expressed as percentage inhibition (PI) values. Samples with a PI value of ≥50% were considered to have a positive result and <50%, a negative result.

### 2.12. Virus Neutralization Test

The virus neutralization test for the FMDV antibody was performed using the methods described in the World Organization for Animal Health (OIE) terrestrial manual [18]. The sera were inactivated at 56 °C for 30 min before testing. Fifty microliters of two-fold serially diluted sera starting from 1/4 dilution were mixed with 50 μL of each virus containing 100 TCID_50_ (50% tissue culture infective dose). After incubation at 37 °C for 1 h, 50 μL of fetal porcine kidney cells (LFBK, supplied by the Plum Island Animal Disease Center (Orient, NY, USA)) were added to each well at 10^6^ cells/mL. The plates were sealed and incubated in an atmosphere of 5% CO_2_ at 37 °C for 2–3 days. The cytopathic effect was observed under a microscope, and the virus-neutralizing (VN) titer was determined using the Spearman–Kärber method [19]. The VN antibody titer was converted to a log_10_ scale for graphical purposes. A titer of 1:45 (1.65 log_10_) or more of the final serum dilution in the serum/virus mixture was considered to indicate a positive result.

### 2.13. Statistical Analysis

The data were analyzed using an unpaired *t* test with the GraphPad Prism software version 5 (GraphPad Software, San Diego, CA, USA), in which a *p*-value of less than 0.05 was considered statistically significant.

## 3. Results

### 3.1. Purification Efficacy of Chromatography Using the Heparin Ligand

**Figure 1:**
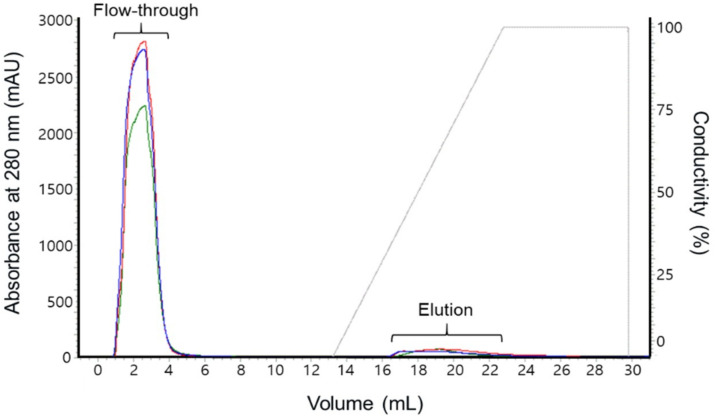
Chromatogram of heparin affinity chromatography. The three strains of the foot-and-mouth disease virus culture supernatant were loaded onto a HiTrap HP heparin column and traced by UV monitoring at 280 nm. The chromatogram shows the fractions obtained during the purification process. The colored lines represent each as follows: blue, O/Boeun/SKR/2017 strain; green, O/Jincheon/SKR/2014 strain; red, Om-O-PanAsia2 recombinant strain; gray, conductivity.

Most of the proteins were obtained in the flow-through fractions of the affinity chromatography using the heparin ligand for all three different virus strains. Small amounts of proteins were detected in the fractions corresponding to 0.5–0.8 M KCl in the gradient elution (Figure 1).

For FMDV O/Boeun/SKR/2017, the total amount of proteins in the flow-through and elution fractions was 61% and 6.9%, respectively (Table 1). For FMDV O/Jincheon/SKR/2014 and Om-O-PanAsia2, the amount of proteins in the flow-through fractions was 61% and 73.8%, respectively. In contrast, the amount of proteins in the elution fractions was 6.9% and 9.1%, respectively, for FMDV O/Jincheon/SKR/2014 and Om-O-PanAsia2.

Meanwhile, 146S was recovered mainly in the elution fractions of the affinity chromatography, regardless of the virus strains (Table 1). The recovery rate of the virus particles for FMDV O/Boeun/SKR/2017, O/Jincheon/SKR/2014, and Om-O-PanAsia2 was 75.4%, 81.5%, and 71.9%, respectively. In contrast, no virus particles were detected in the flow-through fractions.

**Figure 2:**
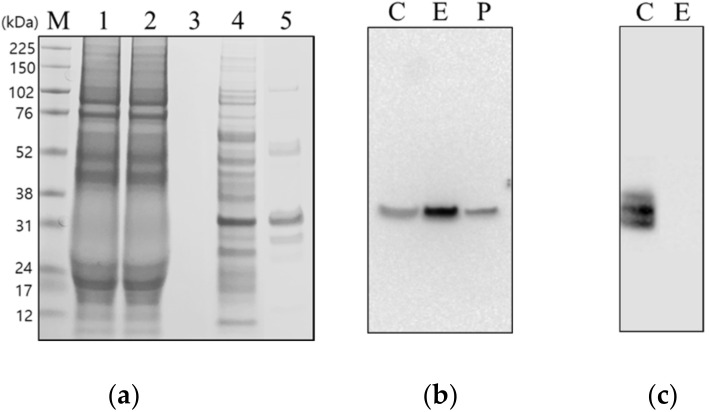
Identification of the purified protein after heparin affinity chromatography. The protein samples of each fraction obtained in the purification process were subjected to sodium dodecyl sulfate–polyacrylamide gel electrophoresis. (**a**) The gel was revealed by Coomassie brilliant blue staining. Lane M, molecular weight marker; Lane 1, foot-and-mouth disease virus serotype O Boeun virus culture supernatant (before column loading); Lane 2, flow-through fraction from the heparin column; Lane 3, washed fraction with the heparin column; Lane 4, eluted fraction from the heparin column (10-fold concentrated); Lane 5, Lane P, positive control (purified FMDV O/Boeun/SKR/2017 by sucrose density gradient ultracentrifugation). Western blot analysis was performed with (**b**) anti-foot-and-mouth disease virus serotype O VP1. structural protein monoclonal antibodies and (**c**) anti-foot-and-mouth disease virus 3B nonstructural protein monoclonal antibodies. Lane C, foot-and-mouth disease virus serotype O/Boeun/SKR/2017 virus supernatant; Lane E, eluted fraction from the heparin column (10-fold concentrated).

Sodium dodecyl sulfate–polyacrylamide gel electrophoresis (SDS-PAGE) of the chromatographic fractions revealed that there were no distinct bands corresponding to the main capsid proteins, such as VP1–VP3, in the crude samples prior to application to the column. The elution fractions only showed apparent capsid protein bands around 30 kDa, which was the same as that of the positive control (purified FMDV O/Boeun/SKR/2017 by sucrose density gradient ultracentrifugation) (Figure 2a). While the crude sample prior to chromatography showed 3AB bands around 30 kDa, no band was observed at the chromatographic elution fraction via the Western blot analysis (Figure 2b).

**Figure 3:**
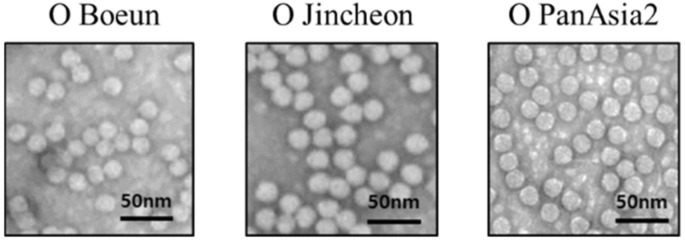
Morphological observation on a transmission electron microscope. The heparin affinity column purified serotype O foot-and-mouth disease viruses were negatively stained and analyzed for morphology. The bars represent 50 nm.

As shown in Figure 3, there was an intact typical FMDV particle with a diameter of 25–30 nm on electron microscopy.

### 3.2. Immunogenicity of the Experimental Vaccine Prepared with Heparin Affinity Column-Eluted Antigens

**Figure 4:**
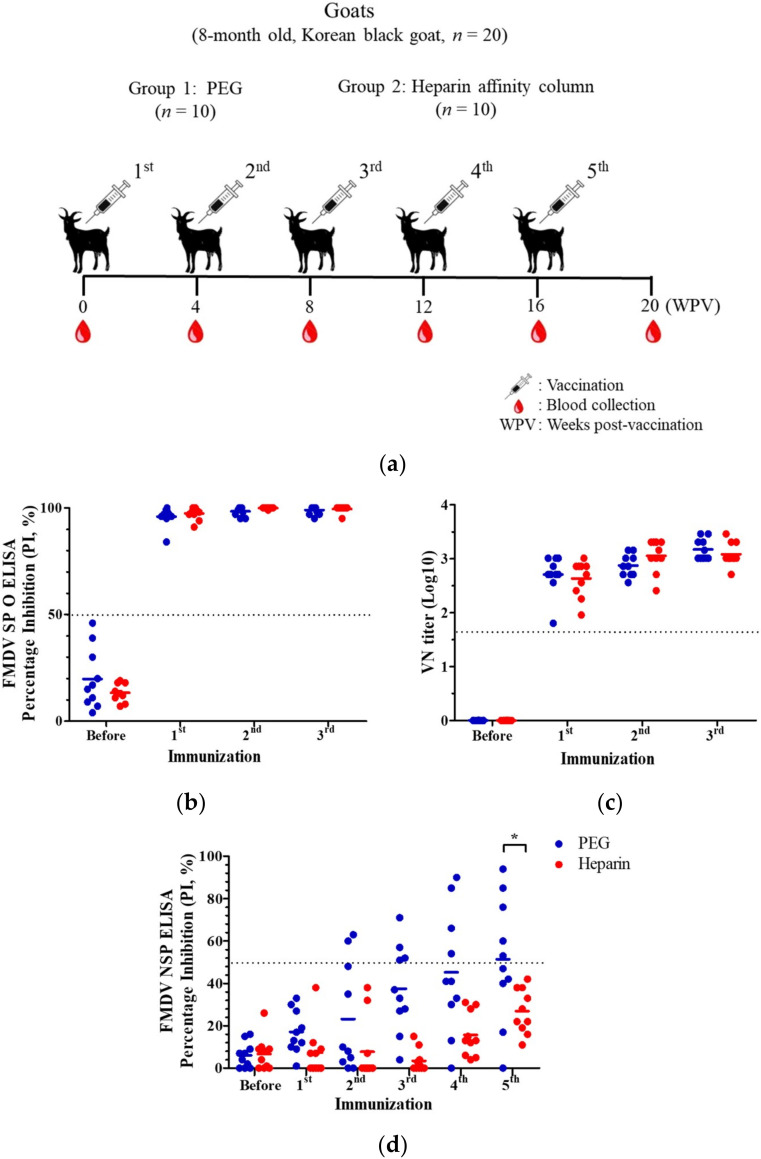
Antibody responses elicited by immunizations with polyethylene glycol (PEG)-treated and heparin affinity column purified serotype O foot-and-mouth disease (FMD) Boeun/SKR/2017 vaccines. (**a**) Scheme of the animal experiment. Twenty goats were vaccinated five times at a four-week interval with PEG-treated (*n* = 10) and heparin affinity column purified (*n* = 10) FMD vaccines. Blood samples were collected every four weeks after vaccination. (**b**) The antibody titers of the structural proteins were measured via enzyme-linked immunosorbent assay (ELISA). The antibody values were expressed as percentage inhibition (PI) values. A PI value of ≥50% (dotted line) was considered to indicate positivity. (**c**) The homologous virus-neutralizing (VN) antibody titers were measured using the virus neutralization test. The VN titers were expressed as a log_10_ value. A titer of ≥1.65 log_10_ (dotted line) was considered to indicate positivity. (**d**) The antibody titers of the nonstructural proteins were measured via ELISA. The antibody values were expressed as PI values. A PI value of ≥50% (dotted line) was considered to indicate positivity. The full blue and red lines represent the mean PI values in each group. A significant difference in the mean values between the two groups in the fifth vaccination was observed. * *p* < 0.05.

The experimental vaccines made with the heparin column-eluted antigens were prepared to evaluate the immunogenicity of these samples in the goats. Twenty goats were vaccinated five times at four-week intervals (Figure 4a). The vaccine was comparable to the PEG-treated vaccine in terms of the SP antibody titers. The PI values of all goats were above 50% from the first immunization and were more than 90% with the booster immunization (Figure 4b).

The VN antibody titers for the homologous virus (O/Boeun/SKR/2017) were above 1:45 (1.65 log_10_) for all goats after the first vaccination, and the subsequent titers were above 1:100 (2.00 log_10_) with the booster vaccination. The titers were the same as those of the PEG group (Figure 4c).

For the NSP antibody, the PEG group was positive for two, four, four, and five heads after the second, third, fourth, and fifth vaccinations, respectively (Figure 4d). Meanwhile, none of the goats in the heparin affinity column group showed positivity for the NSP antibody even after the fifth vaccination. In addition, the heparin affinity column group showed a mean PI value of 26.9% compared with more than 50% in the PEG group after the fifth vaccination. The heparin affinity column group had a significantly lower value (*p* = 0.0226) of mean PI value than the PEG group after the fifth vaccination.

## 4. Discussion

In general, the PEG method is widely used for the concentration and purification of the FMDV; however, some NSPs remain, which is considered a problem. Thus, several studies have been performed to purify the FMDV using chromatography [20,21,22,23,24].

The chromatographic method using gel columns cannot be applied to large-scale production owing to its long working time and limited amount of applicable sample [21]. Conversely, affinity columns have been used in large-scale bioprocessing for nearly 40 years and are applied in the downstream processing of various serotypes of human biopharmaceuticals [25].

Previous studies also introduced heparin affinity chromatography for FMDV purification, which showed a large deviation in the 146S recovery from 40% to 90% [23]. This proved that the application conditions differ depending on the type of FMDV strain used. Importantly, the results of evaluating vaccine purity, one of the major test factors for determining the quality of FMD vaccines, were not presented in that study.

In another study, FMD vaccine antigens were purified with a 75% recovery rate as a result of combining a gel column and a hydrophobic interaction column; however, only one FMDV strain was applied. In addition, the immunogenicity of antibody induction against SPs and NSPs after vaccination was not evaluated [20].

Therefore, in this study, the conditions for purifying various strains of serotype O FMDV developed as FMD vaccine candidates in the Korea, via heparin affinity chromatography, were established. In addition, an experimental vaccine was prepared with the heparin column purified antigen and repeatedly immunized to goats to evaluate the production of NSP antibodies as well as SP antibodies. For confirming the removal of NSPs, 3AB was detected as a representative because it is the most reliable indicator [10].

The FMDV capsid proteins that were not visible before column injection were clearly observed as major bands in the elution fraction, indicating that virus purification was effectively performed. Conversely, in the SDS-PAGE result of the eluted fraction using a heparin column reported by other researchers, the capsid protein of the FMDV was not identified.

As shown in this study, the chromatographic method used was more effective than the PEG-treated method for removing NSPs. NSPs were not detected in the heparin affinity column purified antigen but remained in the PEG-treated antigen in a previous study [10].

The FMDV is known to maintain high immunogenicity when maintained as only intact virus (146S) particles. Therefore, in the process of FMD vaccine antigen production, it is important to recover the 146S particles, including the RNA genome, instead of the capsid-dissociated VP1 subunit protein [26]. From this point of view, previous studies reported that 146S particle recovery varied significantly from 40% to 90%, whereas we observed 146S particle recovery rates over 70% for all three FMDV strains. The antigen yield in this study was much higher than that in the previous study using the same heparin column, which showed 40% recovery of the FMDV and 53% recovery of the porcine reproductive and respiratory syndrome viruses [23,27]. Even our virus strains showed different antigen recovery. It is very difficult to pinpoint the reason for the difference in the antigen recovery. It might due to the different physical characteristics of each FMDV. It remains to be found which physical feature plays the main role in the affinity.

Based on the result that the FMDV was more purified via heparin affinity chromatography than the conventional PEG treatment method, it was necessary to evaluate its immunogenicity in animals by vaccination. To this end, the FMD vaccine antigen prepared via heparin chromatography was adjusted to 15 µg per dose, as determined in a previous study [10], mixed with an adjuvant, and immunized to goats three times to evaluate the antibody levels against SPs and NSPs.

In the SP ELISA and virus neutralization test, both vaccine groups showed the same pattern of antibody levels. This indicates that there was no difference in the induction of protective antibodies against the FMDV even when antigens were further purified by chromatography in comparison with the PEG method. This was a sufficiently predictable result, as shown in a previous report [24].

Another important factor in evaluating FMD vaccines is vaccine purity. According to the OIE manual, the FMD vaccine is acceptable if fewer than two out of eight cattle are positive for NSP antibodies after being vaccinated twice [18]. In this study, goats were employed rather than cattle for the detection of NSP antibody as a vaccine purity test. This was based on the preliminary data in our institute that goats produced NSP antibodies more rapidly and sensitively than cattle in repeated vaccination experiments (data not shown). The PEG-treated vaccine yielded a positive result after the second vaccination, while the vaccine made from a sample purified via heparin affinity chromatography did not induce positivity for NSP antibodies even after the fifth vaccination. In addition, the average PI value of the PEG-treated vaccine increased over 50%, whereas that of the vaccine purified via heparin affinity chromatography remained below 50% regardless of the number of immunizations. Taken together, we demonstrated that the vaccine produced in this study is a higher-purity FMD vaccine than the conventional PEG-treated vaccine.

We reported that NSPs were removed from FMDV culture supernatant by chloroform addition [10]. The immunogenicity of FMD vaccine prepared by this chromatography and chloroform were almost the same against structural and nonstructural proteins (Figure S1). However, total protein concentration of them was different. The chloroform treated vaccine antigen still contains large amount of unwanted cellular protein contaminants compared with heparin affinity column purified vaccine antigen. That is why we established chromatography as an additional purification method. This is the same situation for other FMD vaccine manufacturers that had several methods such as PEG and chromatography in case of emergency situation of further removal of NSP and other cell-derived proteins.

## 5. Conclusions

This study is the first to evaluate FMD vaccine purity by repeatedly immunizing animals with a vaccine prepared via heparin affinity chromatography. This result would be an important groundwork for advanced FMD vaccine manufacturing in the near future.

## Figures and Tables

**Table 1:** Purification efficacy by the HiTrap HP heparin column.

Strain	Step	FMDV Concentration (μg/mL)	Protein Concentration (μg/mL)	Total Protein (%)	FMDV Recovery (%)
O/Boeun/SKR/2017	Crude	27.6	5052.5	0	100
	Flow-through	ND	3084.5	61.1	-
	Elution	20.8	347.0	6.9	75.4
O/Jinchoen/SKR/2014	Crude	16.2	5211.7	0	100
	Flow-through	ND	3672.8	70.5	-
	Elution	13.2	357.1	6.9	81.5
Om-O-PanAsia2	Crude	30.0	5987.5	0	100
	Flow-through	ND	4421.2	73.8	-
	Elution	21.6	546.0	9.1	71.9

FMDV, foot-and-mouth disease virus; ND, not detected.

## Supplementary Materials

The following are available online at https://www.mdpi.com/1999-4915/12/12/1405/s1, Figure S1: Antibody responses elicited by immunizations with chloroform treated and heparin affinity column purified serotype O foot-and-mouth disease (FMD) Boeun/SKR/2017 vaccines.
