# Supplementary material for: Application of Heparin Affinity Chromatography to Produce a Differential Vaccine without Eliciting Antibodies against the Nonstructural Proteins of the Serotype O Foot-and-Mouth Disease Viruses
Source: Viruses. 2020 Dec 7;12(12):1405. doi: 10.3390/v12121405 (PMC7762290; doi:10.3390/v12121405)
Supplement: Supplementary file 1 [file viruses-12-01405-s001.pdf]

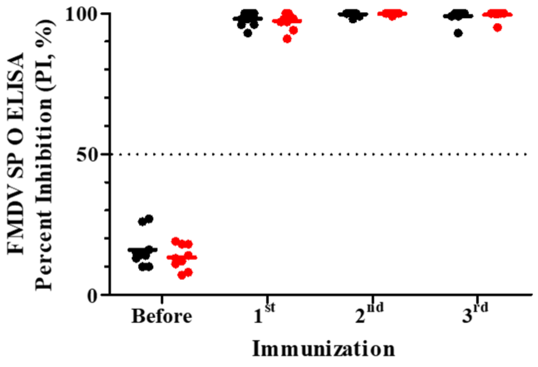

(**a**)

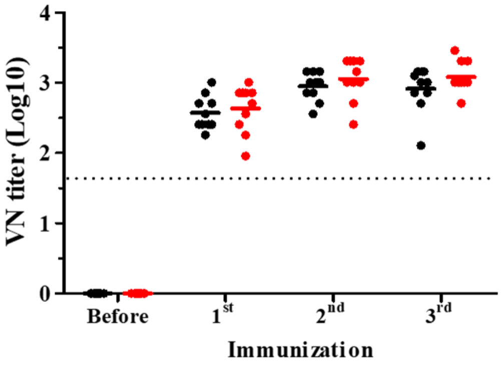

(**b**)

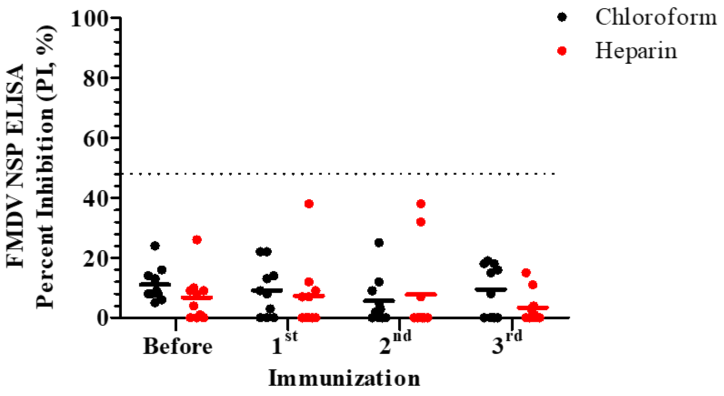

(**c**)

Figure S1. Antibody responses elicited by immunizations with chloroform treated and heparin affinity column purified serotype O foot-and-mouth disease (FMD) Boeun/SKR/2017 vaccines. A total of 20 goats were vaccinated three times at a 4-week interval with chloroform treated (n = 10) and heparin affinity column purified (n = 10) FMD vaccines. Blood samples were collected every 4 weeks after vaccination. (**a**) The antibody titers of the structural proteins were measured via enzyme-linked immunosorbent assay (ELISA). The antibody values were expressed as percentage inhibition (PI) values. A PI value of ≥ 50% (dotted line) was considered to indicate positivity. (**b**) The homologous virus-neutralizing (VN) antibody titers were measured using the virus neutralization test. The VN titers were expressed as a $\log_{10}$ value. A titer of ≥ 1.65 $\log_{10}$ (dotted line) was considered to indicate positivity. (**c**) The antibody titers of the nonstructural proteins were measured via ELISA. The antibody values were expressed as PI values. A PI value of ≥ 50% (dotted line) was considered to indicate positivity. The full black and red lines represent the mean PI values in each group.
